# Physical activity, nutritional status, and dietary habits of students of a medical university

**DOI:** 10.1007/s11332-016-0285-x

**Published:** 2016-06-13

**Authors:** Bogna Grygiel-Górniak, Andrzej Tomczak, Natalia Krulikowska, Juliusz Przysławski, Agnieszka Seraszek-Jaros, Elżbieta Kaczmarek

**Affiliations:** Department of Rheumatology and Internal Diseases, Poznan University of Medical Sciences, Poznań, Poland; Department of Bromatology and Human Nutrition, Poznan University of Medical Sciences, Poznań, Poland; Józef Piłsudski University of Physical Education in Warsaw, Faculty in Biała Podlaska, Biała Podlaska, Poland; Department of Bioinformatics and Computational Biology, Poznan University of Medical Sciences, Poznań, Poland

**Keywords:** Nutritional habits of students, Anthropometric measurement, IPAQ

## Abstract

Nutritional habits and physical activity influence the health status of young adults. In this study, we engaged a group of 151 students from a medical university (90 female and 61 male subjects). Anthropometric parameters, dietary habits (a 7-day dietary recall), and level of physical activity were measured. It was found that the daily food rations of female (F) and male (M) students were improperly balanced and characterized by high amount of total and animal protein, phosphorus, vitamin A, cholesterol, and insufficient intake of carbohydrates, dietary fiber, and vitamin C. Female subjects consumed low amounts of total fat and calcium. The intake of protein (total and animal), fat, phosphorus, and cholesterol correlated with higher body mass. The physical activity of the students was found to be higher than the average physical activity of the European Union populations, and a general tendency of lowering level of physical activity with age was observed. Students with the highest level of physical activity (MET > 1500) consumed lower amounts of simple carbohydrates (galactose and saccharose) when compared to students with lower physical activity (MET < 600, *p* < 0.05). Therefore, this study concluded that the dietary habits should be modified to prevent the development of diet-dependent diseases. Various forms of physical activity should be proposed to students and they should be encouraged to participate in high level of physical activity so as to promote good health status.

## Introduction

Recently, many studies have suggested that lifestyle changes in adolescents and young adults are necessary because of the increased tendency of various adverse health outcomes not otherwise typical for their age, including hypertension, dyslipidemia, and metabolic syndromes [1]. Proper dietary behavior and adequate physical activities reduce the risks of the aforementioned diseases. Many recent studies have underlined the risks of excessive energy intake and sedentary lifestyle in young adults, which can be associated with the increased prevalence of dyslipidemia, obesity, and cardiovascular diseases (CVD) [2]. Particularly, sedentary lifestyle (e.g., playing computer games) is associated with unhealthy snacking patterns, including low intake of fruits and vegetables and overconsumption of energy and fat [2, 3]. Because the level of physical activity influences on physical condition, health status, and quality of life, it is important to undertake physical activity of different intensities until the age of 25–30 to maximize the development of motor skills and physical fitness [4, 5]. However, these recommendations are poorly realized. Taking into consideration the abovementioned criteria, in our study, we attempted to estimate the relationship between nutritional status, dietary habits, and level of physical activity in a group of women and men studying at the Faculty of Pharmacy at Poznan University of Medical Sciences focusing on the prophylaxis of non-communicable diseases such as dyslipidemia, obesity, and osteoporosis.

## Experimental procedures

In this study, 151 students participated from the Pharmacy Faculty of Medical University. All students signed a written consent form. Basic anthropometrical measurements including body mass, height, and waist and hip circumferences were measured; height and waist and hip circumferences were measured using a flexible measuring tape (measurement precision 0.1 cm) and body mass was weighed using a professional body-weight measuring machine (measurement precision 0.1 kg, SECA device). Body mass index was calculated as weight/height squared (kg/m^2^) and waist-to-hip ratio (WHR) as the proportion of waist-to-hip circumferences [6]. The Bodystat 1500 analyzer (BIA method) was used to analyze the body composition of the students (including body fat and lean body mass with measurement precision 0.1 % of total body mass). Because hydration/dehydration processes influence on bioelectrical impedance results, all students were instructed to keep a normal diet and to avoid strenuous exercises for minimum of 3 days before body composition measurement. Students underwent test procedures between 8.00 and 9.30 am in a state of normal hydration (no exercise or alcohol/caffeine consumption in the preceding 12 h and no eating or drinking in the preceding 3–4 h).

**Fig. 1:**
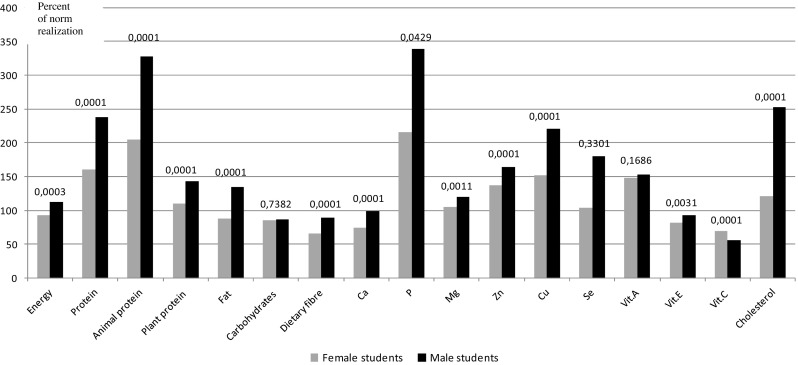
The degree of norm realization on recommended level and nutritional recommendation for energy and select dietary components in female and male group of students in the aspect of cardiovascular diseases prophylaxis. *Ca* calcium, *P* phosphorus, *Mg* magnesium, *Zn* zinc, *Cu* copper, *Se* selenium, *Vit.A* vitamin A, *Vit.E* vitamin E, *Vit.C* vitamin C

The food intake was assessed by the method of 24-h dietary recall of 7 days [7]. Each participant was trained by a dietician and the participants were instructed in detail on what portions have to be consumed and how to record them (they were instructed to weigh every servings and record it; moreover, a special table with the amount of portions to be consumed and a colored nutritional atlas representing the portion sizes of various servings were also used to make the analysis more accurate). All self-reported dietary records were checked and analyzed by the dietician and every questionable recording was once again evaluated together with the students. The results of the questionnaire were analyzed using computer databases stored in Microsoft Access 2000 [8], which were prepared on the basis of the tables of composition and nutrition value of food products [9]. The energy and nutrients intake recommended by the National Food and Nutrition Institute, Warsaw, was considered as a scale to determine the degree to which the recommended energy and nutrients intakes were met [10]. As culinary processes such as boiling decrease the vitamin content, the values expected to be met for vitamin intakes were reduced and are as follows: 25 % for vitamin A, 30 % for vitamin E, and 55 % for vitamin C. The cholesterol and dietary fiber intakes were compared with nutritional prophylaxis recommendation (300 mg/day and 27–42 g/day, respectively) [11]. Because of different recommendations of nutrient intake for F and M, norm realization (NR) was calculated for both groups (Fig. 1). A non-parametric Kruskal–Wallis ANOVA and multiple comparisons of *z*′ values were performed to examine the galactose and saccharose intake in the three groups with different physical activity at a significance level of *α* = 0.05. The level of physical activity was assessed by the International Physical Activity Questionnaire (IPAQ) [12], widely used in Polish population among different age groups characterized by divers education levels and occupations [13, 14]. Generally, in the group with lower physical activity, the individuals took fewer than 600 MET·min/week; in those with sufficient (moderate) physical activity, 600–1500 MET·min/week; and in those with higher physical activity, more than 1500 MET·min/week. Besides physical activity, the intensity and frequency of effort during the week were also assessed [12].

## Results

The age of both female and male groups was similar, but other parameters such as height, body mass, waist circumference, body mass index (BMI), and percent of body fat were determined based on gender (Table 1). The values of waist circumference were within the recommended range and did not exceed 88 cm in F and 102 cm in M [15]. In both genders, the values of waist circumference and WHR were within the health limits (F: WHR < 0.85 and M: WHR < 1.0).

**Table 1:** Anthropometric and nutritional characterization of pharmacy students

Analyzed parameters	Female students *N* = 90	Male students *N* = 61	*p* value
	*X* ± SD	*X* ± SD	
Age (years)	23.0 ± 0.1	23.1 ± 0.2	0.6565
Height (cm)	167.2 ± 0.5	182.0 ± 0.9	0.0001
Body mass (kg)	59.5 ± 0.8	80.1 ± 1.4	0.0001
BMI (kg/m^2^)	21.2 ± 0.3	24.2 ± 0.4	0.0001
Waist circumference (cm)	71.9 ± 0.8	86.5 ± 1.2	0.0001
Hip circumference (cm)	96.1 ± 0.7	98.3 ± 1.1	0.0842
WHR	0.81 ± 0.06	0.88 ± 0.01	0.5003
Body fat (% body mass)	27.6 ± 4.2	19.3 ± 3.6	0.0001
Energy (kcal)	1846.5 ± 42.8	2878.2 ± 124.3	0.0001
Protein (g)	73.8 ± 1.9	121.0 ± 5.7	0.0001
Animal protein (g)	47.0 ± 1.5	83.5 ± 4.7	0.0001
Plant protein (g)	25.2 ± 0.7	36.5 ± 1.8	0.0001
Fat (g)	66.4 ± 1.9	114.5 ± 6.3	0.0001
Carbohydrates (g)	243.2 ± 5.8	343.0 ± 14.3	0.0001
Protein (% energy)	16.2 ± 0.3	17.1 ± 0.4	0.1025
Fat (% energy)	31.5 ± 0.4	35.3 ± 0.7	0.0001
Carbohydrates (% energy)	53.3 ± 0.5	47.9 ± 0.8	0.0001
Dietary fiber (g)	19.7 ± 0.6	26.7 ± 1.3	0.0001
Glucose (g)	6.76 ± 0.4	7.69 ± 0.7	0.1913
Fructose (g)	8.37 ± 0.46	9.69 ± 1.02	0.1729
Galactose (g)	0,66 ± 0,06	0,87 ± 0,17	0.0421
Lactose (g)	9.69 ± 0.43	12.22 ± 1.08	0.0092
Maltose (g)	0.13 ± 0.01	0.21 ± 0.02	0.0001
Saccharose (g)	40.89 ± 1.86	48.74 ± 3.52	0.0362
Starch (g)	146.26 ± 4.34	228.80 ± 10.43	0.0001
Cholesterol (mg)	364.2 ± 15.3	758.8 ± 50.7	0.0001
Calcium (mg)	744.9 ± 31.23	969.6 ± 53.85	0.0002
Phosphorus (mg)	1257.5 ± 36.3	1911.8 ± 98.9	0.0001
Magnesium (mg)	271.4 ± 7.8	385.0 ± 20.4	0.0001
Zinc (mg)	9.37 ± 0.32	15.02 ± 0.73	0.0001
Copper (mg)	1.07 ± 0.03	1.51 ± 0.08	0.0001
Selenium (µg)	47.04 ± 4.42	78.89 ± 3.60	0.0001
Vitamin A (µg)	739.5 ± 54.9	985.5 ± 79.2	0.0002
Vitamin E (mg)	6.48 ± 0.37	9.32 ± 0.91	0.0001
Vitamin C (mg)	42.41 ± 3.92	47.90 ± 9.42	0.5481

Spearman correlation *n* = 151			
Analyzed parameter	R—Spearman	*t* (*N*–2)	*p* value
Protein (g) and body mass (kg)	0.5959	9.0583	0.0001
Animal protein (g) and body mass (kg)	0.5997	9.1488	0.0001
Fat (g) and body mass (kg)	0.5005	7.0575	0.0001
Phosphorus (mg) and body mass (kg)	0.5642	8.3425	0.0001
Cholesterol (mg) and body mass (kg)	0.5339	7.7091	0.0001

*BMI* body mass index, *WHR* waist to hip ratio, *X* arithmetic mean, *SD* standard deviation, *n* number of women

The energy intake was lower than the recommended values in females and higher in males (Fig. 1). Both the protein intake and the percentage of energy from protein were higher than the recommended values in both groups. The consumption of animal protein was two times higher than the recommended values in F and three times higher in M (Fig. 1) and that of fat was lower in F (88.5 %NR) and higher in M (132 %NR). The consumption of carbohydrates and dietary fiber did not fulfill the dietary recommendations (27–40 g/day) in both groups (Table 1; Fig. 1). The amounts of galactose, lactose, maltose, saccharose, and starch were higher in M than in F (Table 1). The starch intake was 60.1 % of the total carbohydrates intake in F and 65.6 % in M, whereas saccharose consumption was 16.8 % of the total carbohydrates in F and 14 % in M (data not shown in the table). The average content of dietary cholesterol exceeded the dietary recommendations (300 mg/day) and was tow times higher in M (*p* = 0.0001).

The intake of macro- and microelements fulfilled or even exceeded the recommended values (Table 1), with the exception of calcium in F, which was only 74.5 % NR (Fig. 1). The intake of phosphorus was twofold higher than the recommended value in F and threefold higher in M, and the intake of vitamin A was higher than the estimated average requirement (EAR). Consumption of vitamin C was lower than the recommended value in both groups and vitamin E was found to be lower in the female group. We also found that the intake of protein (total and animal), fat, phosphorus, and cholesterol correlated with higher body mass (Table 1).

According to the IPAQ criteria, of all respondents, nearly 43 % revealed a high level of physical activity, 45.0 % moderate, and 12.0 % low (Table 2). The percent of women with high and sufficient physical activity was higher than that of men. The detailed analysis of physical activity showed that in the leisure time (recreation and sports), about 61.2 % of respondents undertook intensive physical activity, 31.8 % moderate, and 72.0 % preferred walking (data not shown in tables). As their daily activity, nearly 18.6 % of all respondents performed intensive physical activity, 29.4 % moderate physical activity, and 47.3 % preferred walking. These data showed that students, who participated in sports, usually were involved in intense physical activity, but during recreation time, they preferred light-to-moderate activity such as walking or cycling. In weekdays, nearly 46.0 % of students were observed sitting for more than 6 h daily, while in weekends, nearly 40.3 % of all responders were observed sitting. Results obtained by ANOVA revealed that students with higher level of physical activity consumed lower amount of simple carbohydrates such as galactose and saccharose (Table 3).

**Table 2:** Levels of physical activity examined by criteria IPAQ

Variables	Level of physical activity		
	High MET > 1500	Sufficient MET 600–1500	Low MET < 600
The respondents (*n* = 151)	65 (43.0 %)	68 (45.0 %)	18 (12.0 %)
Women (*n* = 90)	40 (44.4 %)	41 (45.6 %)	9 (10.0 %)
Men (*n* = 61)	22 (36.0 %)	25 (41.0 %)	14 (23.0 %)

**Table 3:** The relation between physical activity and consumption of simple carbohydrates in the group of students

Dietary component	Value of MET (MET·min/week)	*n* (both F and M)	Dietary intake (g) *X* ± SD	*p* value
Galaktoza	MET < 600	33	0.43 ± 0.08	*0.0248 **0.0478 (between MET < 600 and MET > 1500)
	MET = 601–1500	63	0.28 ± 0.06	
	MET > 1500	55	0.23 ± 0.06	
Sacharoza	MET < 600	33	52.56 ± 3.96	*0.0098 **0.0174 (between MET < 600 and MET 600–1500) **0.0173 (between MET < 600 and MET > 1500)
	MET = 601–1500	63	40.22 ± 2.11	
	MET > 1500	55	40.35 ± 2.97	

* *p* value of Kruskal–Wallis test, ** *p* value of multiple comparisons test

## Discussions

Anthropometrical parameters revealed similar age, proper body mass, and BMI values. The results of the body fat content obtained in this study were comparable to that of other studies [16, 17]. The distribution of fat and the values of waist circumference were within the recommended range and thus did not indicate an increased cardio-diabetological risk [15]. Although the use of bioelectrical impendence (BIA) for research is questioned because of its high degree of variability, it is commonly used in many researches [18–20]. Moreover, recent data have shown that BIA methods can also be used in the measurement of other physiopathological conditions like inflammation, hydration, or cell infiltration of fat [18]. Besides this, the measurements made by BODYSTAT 1500 (BIA device) are more accurate than those obtained using the dual frequency BIA device [19] and it is commonly used in many research studies [18–20].

Data analysis of students’ dietary habits showed an improperly balanced daily food rations (DFR) (Table 1). The energy consumption was lower than the recommended values in women and higher in men (88.5 vs 132 % NR, *p* = 0.0003) (Fig. 1). This might be due to the fact that young female subjects usually have a tendency to reduce their energy intake to look thinner, have a tendency to overestimate their weight, and more often reveal an improper body image perception [21].

In both groups, overconsumption of protein was observed and intake of animal protein dominated over the intake of plant protein and this was significantly higher in M (Table 1; Fig. 1). The prospective cohort studies or randomized controlled trials conducted so far underline the supportive influence of protein on bone health [22, 23], and high protein products such as dairy products are also an important source of dietary calcium [24]. However, high amount of protein intake may increase urinary excretion of calcium and decrease bone density showing deleterious effects in early adulthood. This effect is potentiated, particularly, if calcium intake is insufficient [16]. In our study, female students consumed high amount of protein and low quantity of calcium and thus they are at a risk of developing osteoporosis.

An increased intake of fat was observed in male group, but the BMI value was found to be normal, which indicates a high physical activity in this group. In both groups, the percentage of energy obtained from carbohydrates was low and did not reach a minimum of 55 % of energy (most of them should have been derived from polysaccharides) [11]. The intake of all simple carbohydrates (glucose, fructose, galactose, lactose, maltose, and saccharose) was high, whereas the intake of dietary fiber was low. Dietary fiber reveals a positive influence on intestine peristalsis, and the fraction of beta-glucans alters serum lipid profile in a beneficial way [25]. The amount of this component to be consumed should be increased in the DFR of the analyzed groups, as cholesterol intake in these groups was found to be higher than the recommended value and exceeded 300 mg/day.

Intake of selected elements is important for good health status (e.g., bone condition) of young adults [26]. Low intake of calcium in the female group is a risk factor for low bone mineral density, especially when peak bone mass is reached in early adulthood (by the age of 30) [26, 27]. The absorption of calcium depends on the intake of phosphorous. Unfortunately, we observed a high intake of phosphorus in both groups, which exceeded the recommended level and was much higher in male subjects. Indeed, phosphorus is a principal structural element of bone in the form of calcium phosphate salt called hydroxyapatite, but its high dietary intake may contribute to dietary acid load (referred as the renal acid load) and causes bone demineralization [28, 29].

An alarming low intake of vitamins C in both groups and vitamin E in female group was observed (Table 1; Fig. 1). Vitamin C is an antioxidant, which interacts with vitamin E by reducing the tocopheroxyl radical and thus regenerates its native tocopherol [30]. It also enhances intestinal absorption of iron by reducing iron from ferric to ferrous form [31].

Not only the dietary habits but also physical activity influence health (including bone condition) status [27, 32]. In this study, the physical activity of students was higher (Table 2) when compared to the activity during professional duty in Polish population (18.6 % intensive and 29.4 % moderate) [33]. The analysis of MET value (assessed by IPAQ) showed that 43.0 % of all respondents revealed a high level of physical activity (Table 2), which is higher than in the Polish population (33.5 %) and European Union (31.3 %) population [33, 34], but lower when compared to school adolescents (girls—67.7 % and boys—76.2 %) [13]. These data show a general trend of lowering level of physical activity with age. Nearly 12.0 % of F and M students showed low levels of physical activity (low activity is observed in 27.9 % of Polish and 31.0 % of European populations) [33, 34]. This might be probably due to the fact that pharmacy students have a wider knowledge in disease prevention and healthy lifestyle when compared to the normal population.

In this study, we observed an interesting relationship between physical activity and consumption of simple carbohydrates (Table 3). Groups with the highest level of physical activity consumed lower levels of simple carbohydrates (galactose and saccharose) (*p* < 0.05) compared to students with lower level of physical activity. We assume that students with higher physical activity paid more attention to dietary choices and avoided products with high amount of simple sugars.

## Conclusions

This study found that the DFR of F and M students were improperly balanced. The intake of protein (total and animal), fat, phosphorus, and cholesterol correlated with higher body mass. Therefore, it is important to modify the dietary habits of students and monitor them so as to prevent them from the risk of developing dyslipidemia, obesity, or osteoporosis in the future. The study also found that the physical activity of the F and M students was higher than the average physical activity of the Polish adults. Subjects with higher level of physical activity consumed lower amounts of simple carbohydrates. In conclusion, this study emphasizes that various forms of physical activity should be implemented in the schedule of students and they should be encouraged to participate in a high level of physical activity so as to promote good health status.

## References

[1] Anthony D, George P, Eaton CB (2014). American College of Cardiology/American Heart Association; Eighth Joint National Committee Cardiac risk factors: new cholesterol and blood pressure management guidelines. FP Essent.

[2] Chapman CD, Nilsson VC, Thune HÅ, Cedernaes J, Le Grevès M, Hogenkamp PS, Benedict C, Schiöth HB (2014). Watching TV and food intake: the role of content. PLoS One.

[3] Ganesh JS, Rao YY, Ravikumar R, Jayakrishnan GA, Iwasaki M, Preethy S, Abraham SJ (2014). Beneficial effects of black yeast derived 1-3, 1-6 Beta Glucan-Nichi Glucan in a dyslipidemic individual of Indian origin-a case report. J Diet Suppl.

[4] Blair S, Cheng Y, Holder J (2001). Is physical activity or physical fitness more important in defining health benefits?. Med Sci Sports Exerc.

[5] Chakravarthy M, Joyner MJ, Booth FW (2002). An obligation for primary care physicians to prescribe physical activity to sedentary patients to reduce the risk of chronic health conditions. Mayo Clin Proc.

[6] World Health Organization (WHO) (2003) Diet, nutrition and the prevention of chronic diseases. In: WHO technical report series 916, chapter 5, Geneva 12768890

[7] Charzewska J (1998). Instruction of the dietary recall gathering from the last 24 h.

[8] Przysławski J, Walkowiak J, Gertig H, Cichy W, Gajewska B (1998). Nutritional value of daily food rations (DFR’s) taken by cystic fibrosis children. Pediatr Pol.

[9] Kunachowicz H, Nadolna I, Przygoda B, Iwanow K (1998). Tables of nutritious value of food products.

[10] Jarosz M, Bułhak-Jachymczyk B (2013). Recommended values of human nutrition.

[11] WHO (2007) Diet, nutrition Protein and Amino Acid Requirements in Human Nutrition. In: Report of a Joint WHO/FAO/UNU Expert Consultation. Technical reports series 935. WHO, Geneva 18330140

[12] Biernat E, Stupnicki R, Lebiedziński B, Janczewska L (2008). Assessment of physical activity by applying IPAQ questionnaire. Phys Educ Sport.

[13] Bergier B, Bergier J, Paprzycki P (2014). Level and determinants of physical activity among school adolescents in Poland. Ann Agric Environ Med.

[14] Tomczak A (2012). Physical activity of soldiers in the Polish Armed Force’s military administration units and special units. Biomed Hum Kinet.

[15] Expert Panel on Detection, Evaluation, and Treatment of High Blood Cholesterol in Adults (2001). Executive summary of the third report of The National Cholesterol Education Program (NCEP) expert panel on detection, evaluation, and treatment of high blood cholesterol in adults (adult treatment panel III). JAMA.

[16] Heaney RP, Nordin BE (2002). Calcium effects on phosphorus absorption: implications for the prevention and co-therapy of osteoporosis. J Am Coll Nutr.

[17] Olszanecka-Glinianowicz M, Madej P, Zdun D, Bożentowicz-Wikarek M, Sikora J, Chudek J, Skałba P (2012). Are plasma levels of visfatin and retinol-binding protein 4 (RBP4) associated with body mass, metabolic and hormonal disturbances in women with polycystic ovary syndrome?. Eur J Obstet Gynecol Reprod Biol.

[18] Větrovská R, Vilikus Z, Klaschka J, Stránská Z, Svačina Š, Svobodová Š, Matoulek M (2014). Does impedance measure a functional state of the body fat?. Physiol Res.

[19] Simpson JA, Lobo DN, Anderson JA, Macdonald IA, Perkins AC, Neal KR, Allison SP, Rowlands BJ (2001). Body water compartment measurements: a comparison of bioelectrical impedance analysis with tritium and sodium bromide dilution techniques. Clin Nutr.

[20] Datta Banik S, Andrade Olalde AC, Rodriguez L, Dickinson F (2014). The effect of socioeconomic indicators and macronutrient intake rate on body composition in adolescents 12–16 years old in Merida, Yucatan. Anthropol Anz.

[21] Zaccagni L, Masotti S, Donati R, Mazzoni G, Gualdi-Russo E (2014). Body image and weight perceptions in relation to actual measurements by means of a new index and level of physical activity in Italian university students. J Transl Med.

[22] Munger RG, Cerhan JR, Chiu BC (1999). Prospective study of dietary protein intake and risk of hip fracture in postmenopausal women. Am J Clin Nutr.

[23] Schurch MA, Rizzoli R, Slosman D, Vadas L, Vergnaud P, Bonjour JP (1998). Protein supplements increase serum insulin-like growth factor-I levels and attenuate proximal femur bone loss in patients with recent hip fracture. A randomized double-blind placebo-controlled trial. Ann Intern Med.

[24] Johnson-Down L, Ritter H, Starkey LJ, Gray-Donald K (2006). Primary food sources of nutrients in the diet of Canadian adults. Can J Diet Pract Res.

[25] Gunes FE, Bekiroglu N, Imeryuz N, Agirbasli M (2012). Relation between eating habits and a high body mass index among freshman students: a cross-sectional study. J Am Coll Nutr.

[26] Anderson JJ (2001). Calcium requirements during adolescence to maximize bone health. J Am Coll Nutr.

[27] Ilich JZ, Kerstetter JE (2000). Nutrition in bone health revisited: a story beyond calcium. J Am Coll Nutr.

[28] New SA (2002). Nutrition Society Medal lecture. The role of the skeleton in acid-base homeostasis. Proc Nutr Soc.

[29] Remer T, Manz F (1995). Potential renal acid load of foods and its influence on urine pH. J Am Diet Assoc.

[30] Carr AC, Vissers MC (2013). Synthetic or food-derived vitamin C-are they equally bioavailable?. Nutrients.

[31] Gropper SS, Smith JL, Groff JL (2009). Advanced nutrition and human metabolism.

[32] Hoelscher DM, Barroso C, Springer A, Castrucci B, Kelder SH (2009). Prevalence of self-reported activity and sedentary behaviors among 4th-, 8th-, and 11th-grade Texas public school children: the school physical activity and nutrition study. J Phys Act Health.

[33] Piątkowska M (2010) Participation of Poles in physical activity compared to other countries of the European Union. In: Modern methods of research activity, fitness and human performance. AWF Warsaw, University of Physical Education Jozef Pilsudski (in Polish)

[34] Sjöström M, Oja P, Hagstromer M, Smith BJ, Bauman A (2006). Health-en-hancing physical activity across European Union countries: the Eurobarometer study. J Public Health.

